# Social-ecological drivers and dynamics of seagrass gleaning fisheries

**DOI:** 10.1007/s13280-019-01267-x

**Published:** 2019-10-18

**Authors:** Furkon, Natsir Nessa, Rohani Ambo-Rappe, Leanne Claire Cullen-Unsworth, Richard Kazimierz Frank Unsworth

**Affiliations:** 1 Department of Marine Science, Faculty of Marine Science and Fisheries, Hasanuddin University, Tamalanrea Km 10, Makassar, 90245 Indonesia; 2 Sustainable Places Research Institute, Cardiff University, 33 Park Place, Cardiff, CF10 3BA UK; 3 Seagrass Ecosystems Research Group, College of Science, Swansea University, Wallace Building, Swansea, SA2 8PP UK; 4 Project Seagrass, 33 Park Place, Cardiff, CF10 3BA UK

**Keywords:** Coastal zone, Gender, Indonesia, Invertebrates, Seagrass fisheries

## Abstract

Invertebrate gleaning (walking) fisheries are common within intertidal seagrass meadows globally, contributing to the food supply of hundreds of millions of people, but understanding of these fisheries and their ecological drivers are extremely limited. The present study provides a unique analysis of these fisheries using a combined social and ecological approach. Catches contained 34 species and were dominated by Bivalves, Urchins and Gastropods. CPUE in all sites varied from 0.05 to 3 kg gleaner^−1^ hr^−1^, respectively, with the majority of fishers being women and children. Landings were of major significance for local food supply and livelihoods at all sites. Local ecological knowledge suggests seagrass meadows are declining in line with other regional trends. Increasing seagrass density significantly and positively correlated with CPUE of the invertebrate gleaning (*r* = 0.830) highlighting the importance of conserving these threatened habitats. Understanding the complexities of these fisheries, their supporting habitats and their sustainability is important for the support of sustainable coastal livelihoods.

**Electronic supplementary material:**

The online version of this article (10.1007/s13280-019-01267-x) contains supplementary material, which is available to authorized users.

## Introduction

Seagrasses are flowering plants that form ecologically important meadows supporting high biodiversity (Short et al. 2007). Seagrass meadows have a high economic value due to their productivity and the array of ecosystem services (ES) they provide (Costanza et al. 1997; Nordlund et al. 2017; Unsworth et al. 2019). Specifically, a key ES is the provision of shelter, food and nutrients to fish and invertebrate communities, including many species of value for commercial or subsistence fisheries (Unsworth and Cullen 2010; Unsworth et al. 2018).

The complex three-dimensional habitat that seagrasses create results in a diverse and complex food web containing an abundance of macro-invertebrates. Numerous studies have revealed that this animal abundance and diversity tends to be positively correlated with higher seagrass density (Schneider and Mann 1991; Atrill et al. 2000; Unsworth et al. 2007). In addition, macro-invertebrates tend to be more abundant in closed canopy meadows (high seagrass leaf biomass) than open canopy meadows (Vonk et al. 2010). As a result of the high animal abundance and diversity they support, these seagrass meadows create globally important fishing habitats (Nordlund et al. 2018). Coastal seagrass meadows also create excellent fishing habitat because they are generally easy to access, particularly areas that are intertidal (Unsworth and Cullen 2010), and as such these ecosystems are highly exploited by potentially many millions of people globally. Their soft sediment habitat also requires limited gear to exploit, such as small metal tools and buckets.

Gleaning (small-scale collection of invertebrates or other animals from the substrate, usually by hand or with limited, simple gear) has been an important and popular fishing method in intertidal areas from prehistoric times to the present day, due to the easy accessibility of the intertidal zone especially during low tide (Hockey and Bosman 1986; Hockey et al. 1988; Dye et al. 1997; del Norte-Campos et al. 2006). Gleaning can take place in many shallow coastal ecosystems, including reef flats, mud flats, sandy or rocky areas, mangroves and seagrass beds (Nieves et al. 2010). The nature of these fisheries dictates that they are likely characterized by people of low economic activity and provide resources upon which people depend. As a result, these fisheries have a potentially major role in providing food security (Nordlund et al. 2018).

While gleaning is known to be a common human activity in seagrass meadows (Nordlund et al. 2018), there is a lack of specific and detailed information on the intricacies of these invertebrate fisheries which are embedded within complex social-ecological systems (Cullen-Unsworth et al. 2013). In other ecosystems, for example, coral reefs in southern Indonesia, gleaning has been found to result in damage to the coral communities (Tania et al. 2014), however, knowledge on gleaning impacts is limited in a seagrass context. Understanding the complexities of these fisheries, their role in facilitating food security, their drivers and their sustainability is important for the support of effective conservation and for maintaining coastal livelihoods.

The aim of this study was to characterize invertebrate gleaning fisheries in tropical seagrass beds. The study in a region of SE Asia focused on production (species composition, catch volume, CPUE) as well as seagrass condition and its relationship with gleaning production. In addition, this study examined the importance of gleaning to coastal communities, both in general and from a gender perspective.

## Materials and methods

### Study sites

**Fig. 1:**
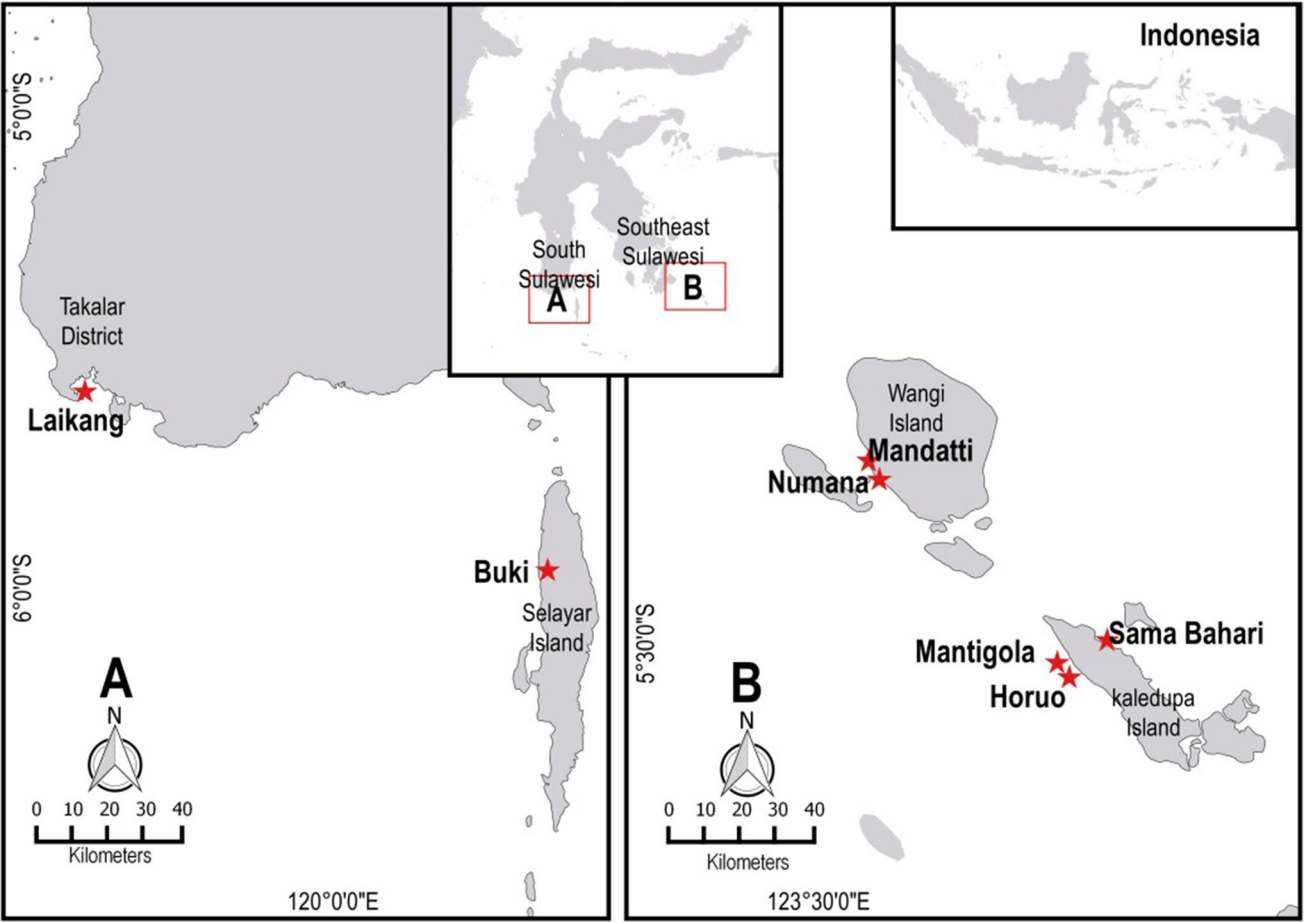
Location of the seven invertebrate gleaning study sites in South and Southeast Sulawesi, Indonesia. Study sites are Laikang and Buki from South Sulawesi and Numana, Mandati, Sama Bahari, Mantigola and Horuo from Southeast Sulawesi. Invertebrate gleaning conducted at all sites

This study was conducted from March 2016 to September 2017 in the intertidal seagrass areas of seven villages around the Island of Sulawesi in Eastern Indonesia. There were five villages spread across the Wakatobi National Park in Southeast Sulawesi (Numana, Mandatti 1, Sama Bahari, Horuo and Mantigola) and two villages in South Sulawesi (Laikang Village and Buki Village) (Fig. 1). All seagrass gleaning areas were fully exposed during low tides and inundated at high tide, although at some sites the seagrass areas extended seawards beyond the intertidal zone. These study sites were chosen due to the presence of extensive seagrass and the known occurrence of intertidal gleaning activity (Table 1).

**Table 1:** Summary characteristics of seven seagrass invertebrate gleaning study sites in SE and South Sulawesi, Indonesia. The villages included four main ethnic groups: Selayar (Buki Village), Makassar (Laikang Village), Wanci (Numana and Mandatti 1 Villages) and Bajo (Sama Bahari, Horuo and Mantigola Villages)

Location		Size (m^2^)	Status	Anthropogenic activities	Substrate
District	Village				
Selayar Island	Buki	54 200	Unregulated	Gleaning activities, boat mooring, set net fishing area, close to coastal villages	Seagrass meadow, sandy
Takalar	Laikang	78 740	Unregulated	Gleaning activities, boat mooring, garbage disposal areas, close to coastal villages, walking area of seaweed farmers	Seagrass meadow, sandy, muddy, rocky, close to mangrove
Wakatobi	Numana	56 430	Local use zone^a^ (limited traditional utilization by the local community)	Gleaning activities, boat mooring, close to coastal villages	Seagrass meadows, sandy
	Mandatti 1	129 600		Gleaning activities, boat mooring, close to harbour	
	Sama Bahari	7180		Set net fishing area	
	Horuo	1340		Gleaning activities, boat mooring, close to coastal villages	
	Mantigola	1340		Gleaning activities, boat mooring, close to coastal villages	

^a^Forestry Ministry Regulation No. p/56, 2006

### Data collection on gleaning activity

Profiles of invertebrate gleaning were constructed based on field surveys, questionnaires and informal interviews with gleaners. Interviews were used to collect data on gleaners (gleaner profiles), invertebrate gleaning methods and gleaner perceptions regarding seagrass meadows (Tables S1 and S2). Gleaner perception data were collected to determine gleaner knowledge regarding the relationships between gleaning activity, animal abundance and seagrass condition. Invertebrate gleaning activities involved both adults and children.

All interviews were semi-quantitative using a variation of a questionnaire previously used in some of these locations (Unsworth et al. 2014) that had been trialled throughout a range of projects (Cullen-Unsworth et al. 2011). Gleaners in the field were sampled haphazardly as we met them. We surveyed and interviewed as many as was possible at all sites. A total of 106 gleaners were interviewed randomly across the study sites (Tables S1 and S2). This is an unbalanced design with respect to numbers of villages, the unbalanced design is a reflection of the ease with which it was possible to interview people as this can vary between sites based on site access and as a result of fishers interest and disinterest in our work. An estimation of the total number of gleaners was obtained through interviews in the field with village elders of leaders.

The field survey on gleaning activity was conducted by accompanying the gleaners for 2–4 h in the field as they collected their catch, this allowed for direct observation of their target fauna, their locality of collection within the seagrass. The gleaners in a given area usually worked in a group and pooled their catches at the end. The number of gleaners and the total time they spent gleaning were recorded allowing us to calculate CPUE as a measure people collecting per unit hour. Species, abundance and catch weight (kg) were also recorded by the taxonomic group for each group of gleaners. Each animal was identified to the lowest possible taxonomic level, and reference specimens were collected in order to confirm identifications using FAO (1978), Indonesian Shell II (Hemmen 1992), sealifebase.org and gastropods.com.

### Seagrass diversity and cover

Seagrass species composition and cover (%) were estimated using a systematic sampling method according to English et al. (1994). Three 100 m line transects were placed perpendicular to the shoreline at each site, 50 m apart. Ten quadrats (50 cm × 50 cm) were deployed along each line transect at 10 m intervals. Within each quadrat, seagrass (%) cover and species composition were determined. Dominancy of each species was estimated based on the coverage (%) of each species in each transect quadrat (Rahmawati et al. 2014).

### Data analysis

Data were tabulated and summarized to produce mean values (with standard deviation) and CPUE (kg gleaner^−1^ h^−1^). The invertebrate gleaning profiles were analysed descriptively, while between village differences in species richness, animal abundance and catch weight were analysed using One Way Analysis of Variance (ANOVA). Significance was evaluated at the 95% confidence level (*α* = 0.05). Correlations between seagrass cover (%) and invertebrate variable (CPUE) across the sites were estimated using Pearson correlation coefficient (*r*) using SPSS (version 16.0). In order to further elucidate differences in species assemblages between gleaners and between sites, multivariate analysis with non-metric multi-dimensional scaling (nMDS) was applied in PRIMER© 6.0 (Clark and Gorley 2006).

## Results

### Gleaning habitat

**Fig. 2:**
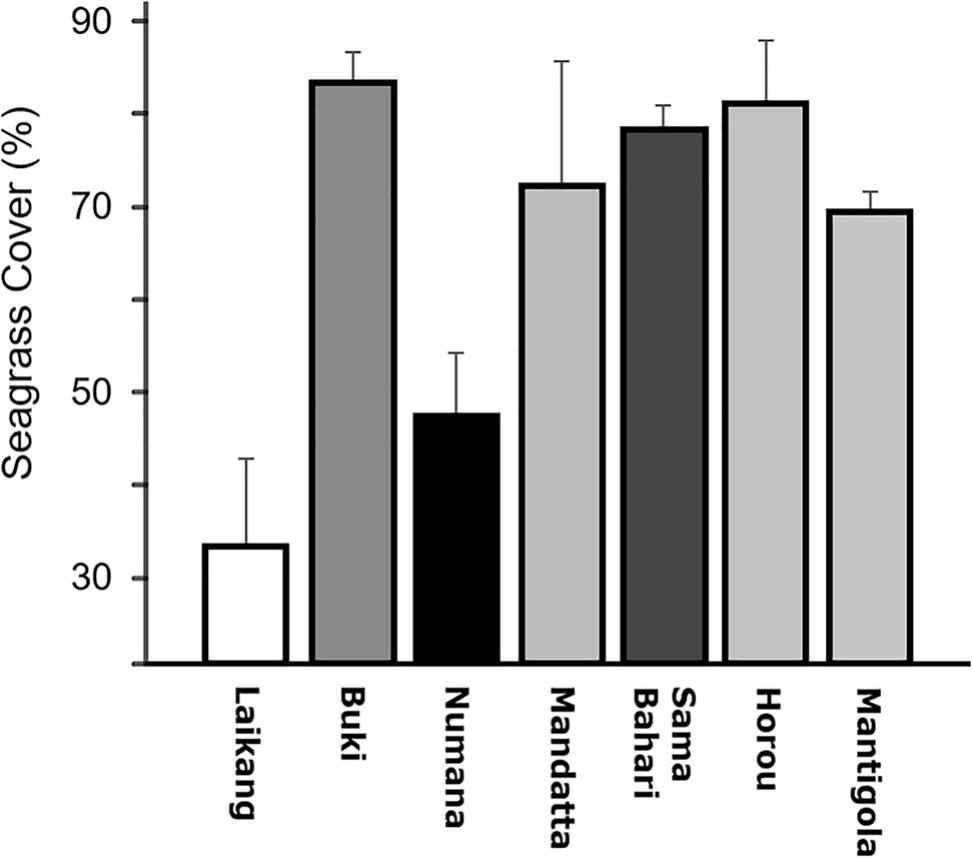
Mean (± SD) seagrass percent cover across seven villages in Sulawesi, Indonesia

Seagrass was the dominant habitat type at all sites but species composition and cover varied between sites (Fig. 2). Buki Village had the highest average seagrass cover (83.3%), while Laikang (35%) had the lowest seagrass cover. Then seagrass cover in Numana (47.5%), Mandatti 1 (72.4%), Sama Bahari (78.2%), Horuo (81.1%) and Mantigola (69.4%). Six seagrass species were identified; *Enhalus acoroides, Thalassia hemprichii, Cyamodocea serrulata, Cyamodocea rotundata, Halodule uninervis, Halophila ovalis and Syringodium isoetifolium*. *Thalassia hemprichii* was the most dominant species at all sites except Laikang.

### Fisher profiles

**Fig. 3:**
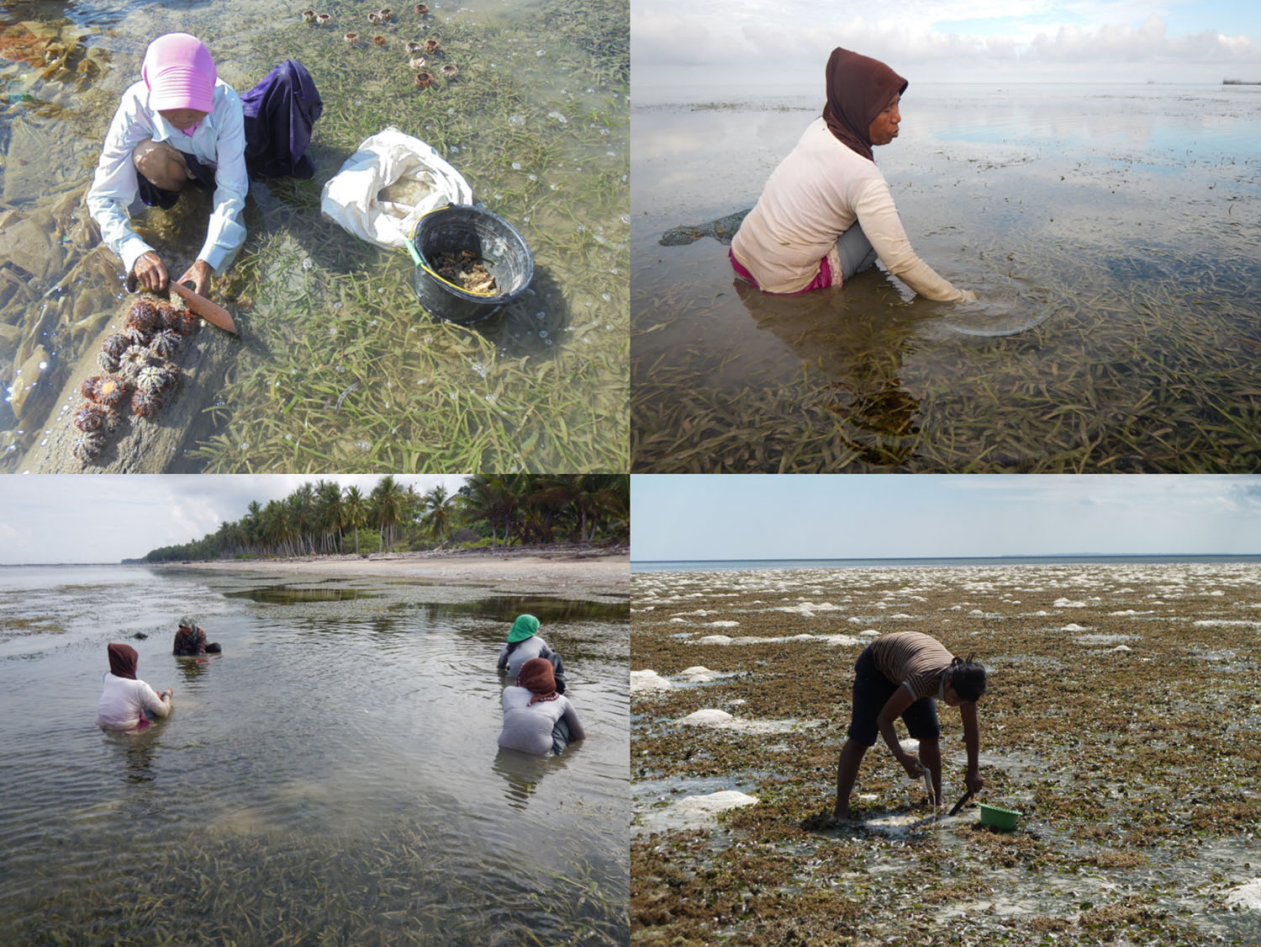
Example gleaning activity on seagrass from throughout SE Sulawesi, Indonesia (Photos: Authors Furkon & Cullen-Unsworth)

Women comprised the largest group of gleaners at all sites (52%), followed by children (31%), while men comprised less than a fifth of all gleaners (17%) (see Appendix) (see Fig. 3). The male gleaners predominantly considered themselves as fishers (11.3%). The non-fishers (88.7%) described their employment status as housewives (*n* = 31), working in or running small businesses (*n* = 10), farmers (*n* = 3), freelance (*n* = 6), teachers (*n* = 2), students (*n* = 37), unemployed (*n* = 4) and civil servants (*n* = 1) (Fig. S1).

Gleaning in Laikang Village was divided into two types, general and specific. General gleaning was called *mattude* in the local language; mostly conducted during the day, all edible species found would be collected. The gleaners only used their bare hands, with plastic bags or buckets in which to put the harvest. Specific gleaning was mostly conducted during the night, and crabs were the only target species; gleaners used additional tools such as gloves and a torch or headlamp.

Gleaning activities in Buki and Sama Bahari villages were limited to general gleaning (locally called *ngatti*–*ngatti*). The gleaners collected all edible animals found, using tools such as a machete or a spear. Gleaning was common in the Wakatobi Archipelago, where local names for gleaning activities included *meti*–*meti* and *tunga* in Wangi–Wangi Island, and *nubba* in the Bajo (sea gypsy) communities of Kaledupa Island (Sama Bahari, Horuo and Mantigola Village). All gleaning activities in Wakatobi were of the general gleaning type.

Gleaners in Buki and Sama Bahari villages mostly preferred to glean as individuals, while at the other five sites most gleaners preferred to work in groups. The majority of gleaners were indigenous to the village where they conducted their gleaning activities, with the exception of Buki, where half of the gleaners came from outside the village.

The utilization of the gleaning catch was similar at six of the sites, where gleaning was primarily for food (subsistence fishing), although some of the catch was also sold to obtain cash income in the two South Sulawesi sites (Buki and Laikang). The exception was Sama Bahari Village in the Wakatobi Islands where gleaning was primarily an income-earning activity, with the majority of gleaners (71%) selling their catch (Fig. S1).

### Species composition of catch

**Fig. 4:**
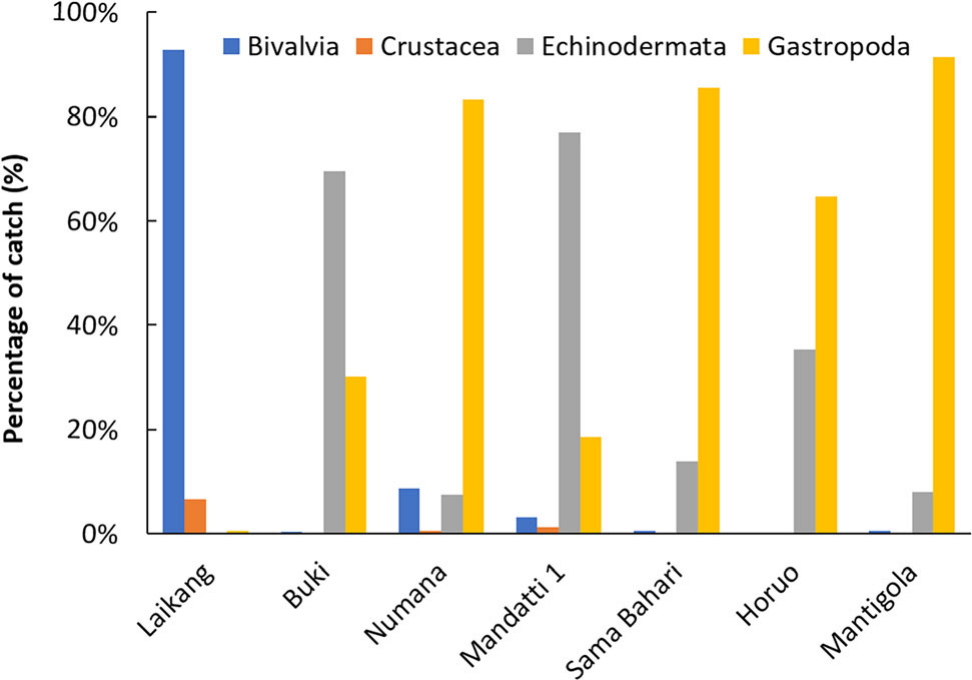
The catch composition (%) during invertebrate gleaning in seagrass at seven villages in Sulawesi, Indonesia

The catch composition recorded during the field survey varied between sites both in terms of the species collected and the number of individuals of each species (Table S3). However, the catch tended to be dominated by three major taxonomic groups: the bivalves (*Gafrarium tumidum*), gastropods (*Canarium urceus*) and echinoderms (*Tripneustes gratilla*), with some crustaceans (*Thalamita sima*) (Fig. 4). The most commonly collected species was *Gafrarium tumidum*. *Anadara antique*, *Gafrarium tumidum* and *Tripneustes gratilla* were the species that were collected commercially. In Laikang, nine species were collected; the most common taxonomic group was bivalves (92.7%), dominated by tumid venus (*Gafrarium tumidum*) (local name *tude kapala bibir*) and asiatic hard clam (*Sinanodonta woodiana*) (local name *tude laccu*). In Buki, 16 species were collected dominated by echinoderms (69.5%), especially *Tripneustes gratilla* (local name *tie*–*tie*) and *Salmacis sphaeroides* (local name *tie*–*tie kalubinting*). In Numana, out of 1880 animals collected, 83.2% were gastropods (dominated by *Canarium urceus*). Echinoderms (77.1%) were the dominant taxon in Mandatti 1 (especially *Tripneustes gratilla* with 289 animals), while gastropods were the most commonly collected taxonomic group in Sama Bahari (85.6%, predominantly *Conomurex luhuanus* with 1300 individual), Horuo and Mantigola (64.62% and 91.41%, respectively, dominated by *Canarium urceus* with 410 and 1563 animals collected, respectively).

### Seasonality

Data collection was spread in an unbalanced design across two seasons (intermonsoon 1 (west to east) and east monsoon) as it was not possible to assess all sites during one season. Laikang, Buki, Numana and Mandatti 1 are included in the Intermonsoon 1 (March–April), while Sama Bahari, Horuo and Mantigola are in East Monsoon (May). Species richness, animal abundance, total catch and number of gleaners tend to be higher in intermonsoon 1 than east monsoon (Fig. 2).

### Community composition of catch

**Fig. 5:**
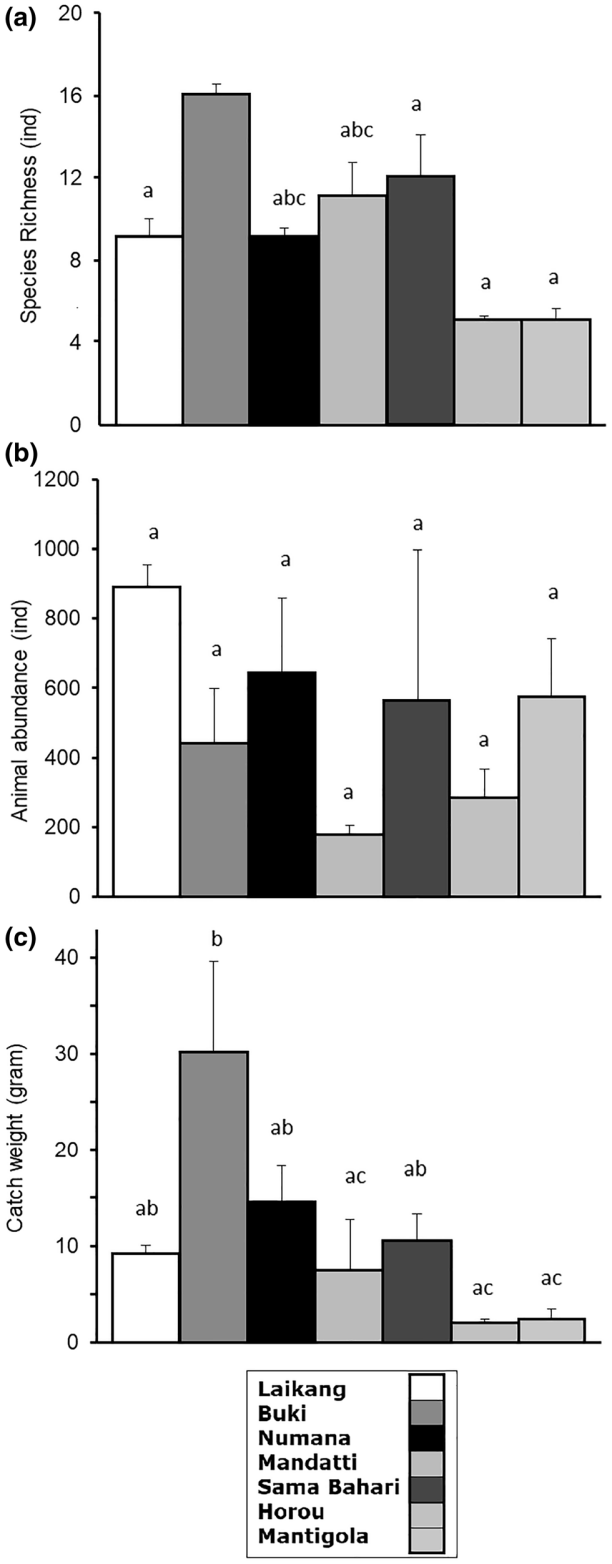
Mean (± SD) catch characteristics **a** Species richness, **b** Animal abundance and **c** Catch weight for invertebrate collected by gleaners in seagrass at seven villages in Sulawesi, Indonesia. Superscripts **a**–**c** are significantly different (*p *< 0.05)

The analysis of invertebrate catch structure included measures of species richness, animal abundance and catch weight, showed that structure varied between and within sites (Table 2). Species richness (*p* = 0.03) and catch weight (*p* = 0.011) were significantly different between sites (*p* < 0.05). Mean invertebrate species richness was higher in Buki Village (16 species) than at any other site (Fig. 5a), specifically with Laikang, Sama Bahari, Horuo and Mantigola. Mean catch weight was also highest (30.07 kg) in Buki Village (Fig. 5c), however, significant differences (*p* < 0.05) were found between Buki, Mandatti 1, Horuo and Mantigola sites. Mean animal abundance was highest in Laikang Village (882 individuals, all species combined) (Fig. 5b), however, the between-site differences were not significant (at *α* = 0.05).

**Table 2:** Analysis of Variance table. Examining differences in species richness, animal abundance and catch weight of gleaning fisheries within and between seven villages in Sulawesi, Indonesia

Source of variance		*df*	Mean square	*F*	*P*
Species richness	Between groups	6	23.5	5.885	0.003
	Within groups	14	4.0		
	Total	20			
Animal abundance	Between groups	6	167 431.4	1.273	0.330
	Within groups	14	131 477.9		
	Total	20			
Catch weight	Between groups	6	2.766E8	4.369	0.011
	Within groups	14	6.331E7		
	Total	20

**Fig. 6:**
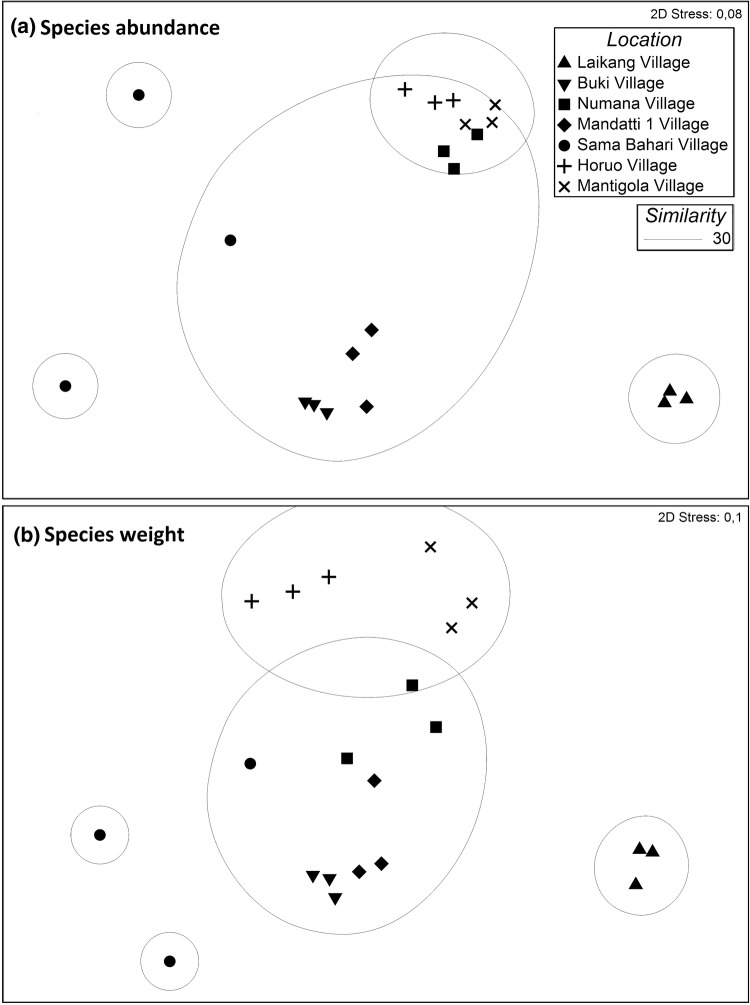
Non-metric MDS scaling configuration with superimposed Bray–Curtis similarity clusters at the 30% level for comparisons of **a** animal abundance and **b** catch weight between from invertebrate gleaning catches on seagrass at seven sites throughout Sulawesi, Indonesia

The multivariate nMDS ordination indicated a distinct separation of invertebrate community structure both between and within the seven sites (Fig. 6). The significance level of this separation was confirmed by an ANOSIM based on animal abundance (*R* = 0.826, *p* < 0.001), and catch weight (*R* = 0.804, *p* < 0.001).

In Laikang, general and specific gleaning were analysed separately. The gleaners (*N* = 106) typically spent 2 to 4 h gleaning, at all sites. The total catch volume, volume gleaner^−1^ and CPUE were highest in Buki and lowest in Laikang.

### Seagrass as a gleaning habitat

**Fig. 7:**
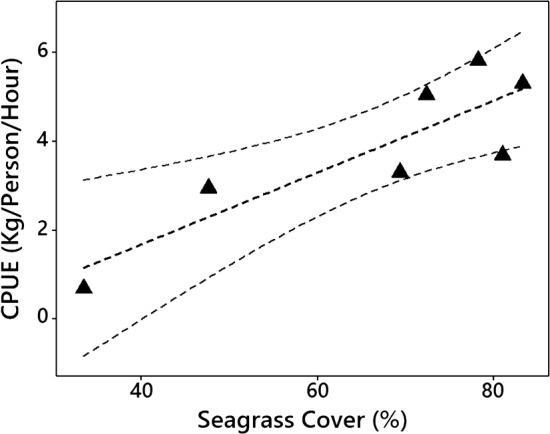
Correlation (showing 95 CI) between seagrass cover and CPUE of invertebrate gleaning landings across seven seagrass sites in Sulawesi, Indonesia. Regression lines: *R*^2^ = 0.83, *p *= 0.021

Overall, the majority of gleaners considered that gleaning activities damage the seagrass meadows by trampling. Just over half of the gleaners (51%) considered that gleaning production was related to the condition of the seagrass meadows, while 6% were unsure and almost half of gleaners (43%) did not think there was a correlation. Our gleaning landing data linked to our habitat data find that CPUE (Catch Per Unit Effort) (Table 3) of invertebrate gleaning in seagrass meadows is significantly and positively correlated with seagrass cover across all sites (Pearson correlation, *r* = 0.83, *p* = 0.021) (Fig. 7).

**Table 3:** Invertebrate gleaning production (± Standard Deviation) for general gleaning (day) and specific gleaning (night) within seven villages of the Southern Sulawesi. Studies conducted from March 2016–September 2017

Village	Type	Intensity (hour)	Number of gleaners	Total catch volume (kg)	Catch volume per gleaner (kg)	CPUE (kg gleaner^−1^ h^−1^)
Laikang	General	3	21	27.04 ± 1.7	1.29 ± 0.3	0.43 ± 0.1
	Specific	2	8	3.06 ± 1.2	0.38 ± 0.03	0.05 ± 0.02
Buki	General	2	16	93.33 ± 17.3	5.83 ± 0.2	2.92 ± 0.6
Numana	General	2	15	37.98 ± 8.3	2.53 ± 0.4	1.27 ± 0.2
Mandatti 1	General	2	12	54.79 ± 4.8	4.56 ± 2.3	2.28 ± 1.1
Sama Bahari	General	4	14	77.07 ± 22.4	5.50 ± 3.1	1.38 ± 0.2
Horuo	General	2	11	41.90 ± 7.8	3.81 ± 0.3	1.90 ± 0.3
Mantigola	General	2	9	28.26 ± 0.7	3.14 ± 0.8	1.57 ± 0.3
Total		19	106	363.43 ± 64.2		
Average		2.37	13.25	45.43 ± 8.02	3.38 ± 0.93	1.48 ± 0.35

## Discussion

The present study provides the first quantitative catch evidence documenting the widespread extent and importance of gleaning activities in seagrass for food supply in SE Asia.

This study provides novel correlative evidence supported by Local Ecological Knowledge (LEK) that increasing seagrass density and condition supports a more productive fishery highlighting the need to protect these threatened systems (Unsworth et al. 2018). By bringing together LEK with in field ecological and fisheries data we’ve been able to more fully understand the status, threats and changes to these fisheries as LEK is known to be strong in this region (Pilgrim et al. 2008) (Fig. 3).

Gleaning fisheries were split into two main forms, general and targeted “specific” gleaning for commercial species. General gleaning for food supply was present in all sites; however at some locations targeted (species specific) gleaning was also conducted. Previous studies in other parts of the Indo-Pacific have documented similar patterns (del Norte-Campos et al. 2006).

Gender was a major factor defining these fisheries with women comprised the majority of general gleaners, outnumbering children and men combined. This provides further evidence to a growing wealth of literature highlighting the important role of women in coastal community provisioning throughout the tropics (Jiddawi and Ohman 2002; Al Rashdi and McClean 2014; Kleiber et al. 2014). Women are found to use gleaning and other nearshore fishing activity as a means of providing protein in household diets, as well as in some cases contributing to household income through the sale of their catch (general and specific gleaning). In many locations, women tend to have a higher level of participation in coastal resources utilization compared to men, particularly for gleaning activities (de la Torre-Castro et al. 2017).

Gleaning at all sites was mostly conducted as a source of food (subsistence). However, at one of the Bajo (sea gypsy) villages, most gleaners viewed their catch as a source of income rather than as a source of food. In the late 1990s, a gleaning fishery for sea cucumbers and other invertebrates, involving mainly women and children as in this study, was one source of income for the communities in this and other nearby villages (Moore 1998). These findings may reflect a need for the mostly landless indigenous Bajo communities to trade to obtain vegetables and rice (or cassava), due to their limited access to land to grow to produce (Cullen-Unsworth et al. 2007).

Most gleaning was conducted at a local level close to gleaners homes, with few outsiders taking part. Gleaners targeted seagrass areas as places to collect the highest abundance of invertebrates. This preference for seagrass is similar to other case studies, where gleaners chose areas with high percentage of seagrass cover as the best places to harvest invertebrates (Nordlund et al. 2010). Thirty-four invertebrate species were recorded in gleaning catches, with substantial variation in species abundance and diversity between seagrass areas. Bivalves, echinoderms and gastropods dominated the invertebrate gleaning catch at all sites; this is not surprising, as the sites offer suitable habitat for these burrowing and suspension-feeding species. Furthermore, bivalves, crustaceans and gastropods are widely reported as animals associated with seagrass meadows (Duarte 2002; Nordlund and Gullstrom 2013; Libres 2015), as are some echinoderms, including *Tripneustes sp*. (Nordlund et al. 2010). Bivalves, in particular, have been reported as comprising a significant proportion of seagrass gleaning catches in other countries, including the Philippines (del Norte-Campos et al. 2006; Nieves et al. 2010) and Mozambique (Nordlund and Gullstrom 2013).

We hypothesize from our findings that selective gleaning, habitat type and habitat structure are major contributory factors to the dominant catch species at each site and the overall catch abundance. This hypothesis is based on the observed significant correlation between seagrass cover and CPUE of these gleaning fisheries, and supported by strong Local Ecological Knowledge (LEK). For example, the tumid venus clam (*Gafrarium tumidum*) was the dominant species, comprising nearly 90% of the catches at one particular locality; while possibly be due to gleaner preference, this dominance could well be a result of the presence of sandy and muddy substrate adjacent to mangrove stands, similar to the habitat of this bivalve in New Caledonia (Baron and Clavier 1992). The sites where the urchin (*Tripneustes gratilla*) was the main target species had the highest percentage cover of seagrass (> 80%). The high abundance of this urchin may reflect known positive correlations between seagrass health and *T. gratilla* abundance (Vonk et al. 2008; Lyimo et al. 2011; Silahooy et al. 2013). Habitat structure and composition are considered to be the primary factors influencing the dominance of *Canarium urceus* (Levinton 2009), which favours muddy and sandy areas (Won et al. 2012) with seagrass and algae cover (Vroom and Braun 2010; Superales et al. 2016). This gastropod was the dominant species collected at three sites where the observed characteristics of seagrass meadows used for gleaning correspond to the reported habitat preferences of *C. urceus*.

The present research indicates that species diversity in gleaning catches might also be related to habitat structure. The highest number of species (16) was collected where seagrass cover was also highest. Conversely, the number of species collected was lowest (5) in Mantigola, with the second lowest seagrass cover. This is in line with findings that healthy seagrass meadows sustain higher species richness than unvegetated habitats (Edgar 1990; Bostrom and Bonsdorff 1997), and that species number can be significantly higher in areas with high seagrass cover than in those with low seagrass cover (McCloskey and Unsworth 2015).

Our observed correlation between seagrass cover and CPUE supports the theory that increasing seagrass habitat complexity and resources support more abundant and diverse fauna worldwide (McCloskey and Unsworth 2015). Variations in average seagrass cover might be related to several anthropogenic activities. For example, at the site in South Sulawesi (Laikang) where the seagrass cover was lowest, general gleaning production and CPUE for general and specific gleaning combined were also low while both gleaning effort and other anthropogenic impacts were high. Potentially damaging activities other than invertebrate gleaning included boat mooring, garbage disposal and trampling by seaweed farmers. There exists increasing evidence that such damaging activities are widespread throughout the Indonesian archipelago (Unsworth et al. 2018). Our data indicate that this loss may be having major negative impacts upon the nation’s intertidal invertebrate fisheries. Eckrich and Holmquist (2000) found that *Thalassia testudinum* beds experienced reductions in seagrass cover and animal density due to trampling activities on these seagrass beds. Nordlund et al. (2010) reported that increases in the number of invertebrate gleaners caused declines in seagrass health and gleaning production.

The present assessment provides the first quantitative assessment of seagrass gleaning fisheries in the SE Asia; given the extensive nature of this activity, and the clear links between seagrass gleaning fisheries, human livelihoods and poverty, a more detailed understanding is required. This sort of widespread fishery is too big to ignore, not just in Indonesia but across the region. Our current dataset requires expansion through time and space to elicit the drivers of the productivity of these fisheries.

## Conclusion

Invertebrate gleaning is an important fishing activity for coastal communities in SE Asia providing a source of food and livelihood income. In the present study, we find evidence for the value of seagrass in supporting these fisheries and the negative effect of declining seagrass habitat on fishery productivity. Importantly women were found to be the dominant group leading this fishing activity, supporting the growing wealth of literature recommending the greater inclusion of women into fisheries management. Given the increasing loss of seagrass meadows throughout the region, our study highlights why such losses may be having major negative effects upon the regions’ fisheries.

## Electronic supplementary material

Below is the link to the electronic supplementary material.

Electronic supplementary material 1 (PDF 508 kb)
