# Supplementary material for: Social-ecological drivers and dynamics of seagrass gleaning fisheries
Source: Ambio. 2019 Oct 18;49(7):1271–81. doi: 10.1007/s13280-019-01267-x (PMC7190608; doi:10.1007/s13280-019-01267-x)
Supplement: Supplementary file 1 — Electronic supplementary material 1 (PDF 508 kb) [file 13280_2019_1267_MOESM1_ESM.pdf]

Ambio

Electronic Supplementary Material

Title: **Social-ecological drivers and dynamics of seagrass gleaning fisheries**

Furkon, Natsir Nessa, Rohani Ambo-Rappe, Leanne Claire Cullen-Unsworth, Richard Kazimierz Frank Unsworth

Table S1. Gleaner Composition by Site (n=106)

Location		Adult		Children		Total sample	Estimated total gleaners
District	Village	Male	Female	Male	Female		
Selayar Island	Buki	2	14	0	0	16 (21%)	77
Takalar	Laikang	7	12	5	5	29 (34%)	85
	Numana	1	11	0	3	15 (21%)	70
	Mandatti 1	2	5	5	0	12 (17%)	69
Wakatobi	SamaBahari	6	6	0	2	14 (23%)	61
	Horuo	0	3	1	7	11 (22%)	50
	Mantigola	0	4	0	5	9 (36%)	25
Total		18	55	11	22	106 (100%)	437

Table S2. Sample questionnaire used to interview a gleaner in the field (translated from Indonesian)

Profiles	
What is your name?	
Gender	Men
	Women
Where is your home?	
What is your highest educational attainment?	Elementary school
	Junior high school
	Senior high school
	Higher education
	None
What do you do for a living?	
What is your motivation for gleaning?	To eat
	To sell
How do you glean?	Individually
	In a group
Perception	
Are seagrasses similar to seaweeds?	Yes
	No
	No idea
Can gleaning damage the seagrass?	Yes
	No
	No idea
Is there a relationship between seagrass condition and gleaning catch?	Yes, (positively correlated)
	No
	Uncertain
	No idea

Fig. S3. Gleaner composition by site (n=106); (a) Gleaning pattern, (b) Catch utilization, and (c) Employment

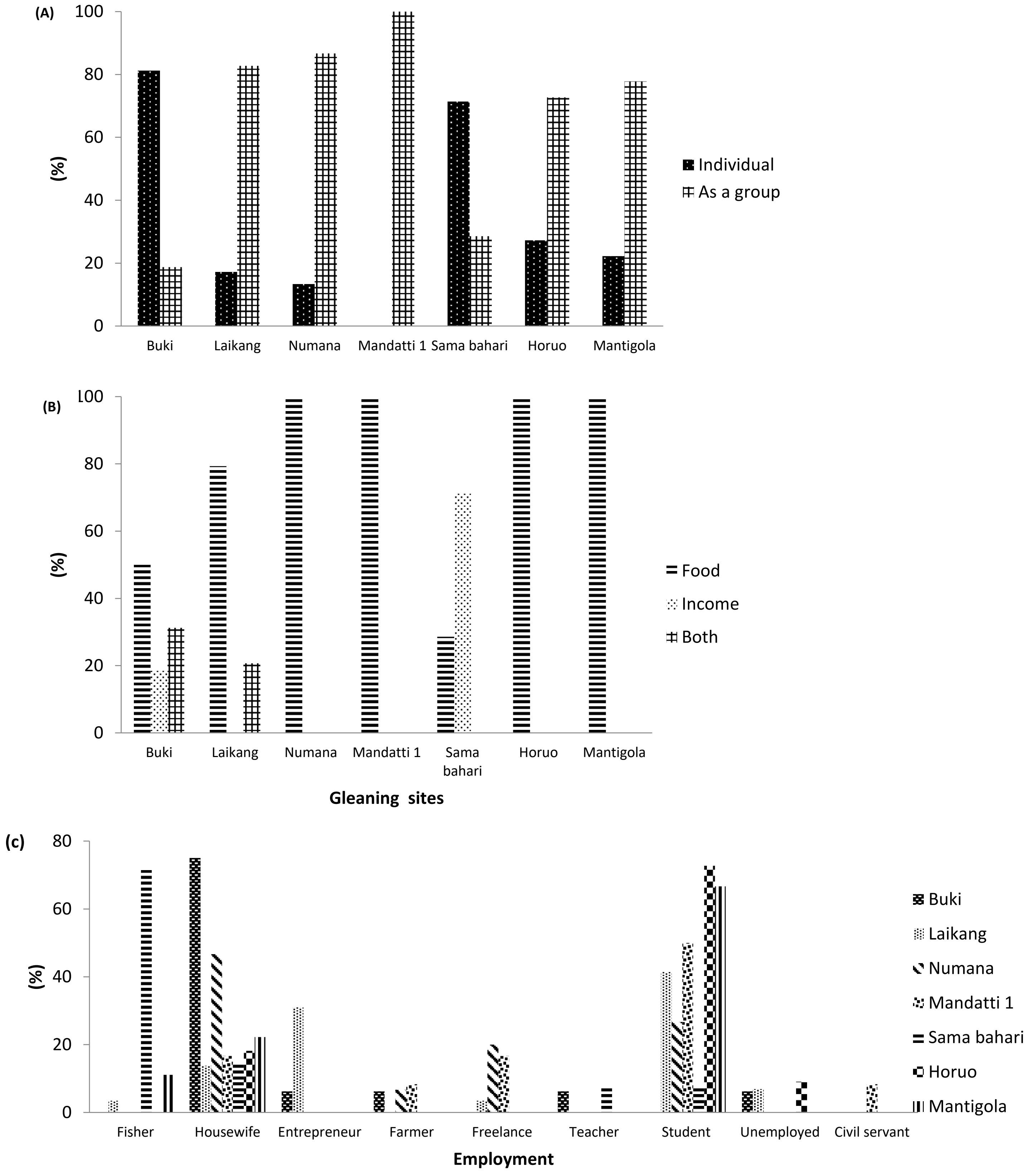

Table S4. Animals collected by invertebrate gleaners by species and site

Taxa	Species	Village						
		Laikang	Buki	Numana	Mandatti 1	Sama Bahari	Horuo	Mantigola
Bivalves	Anadara antique	8		163	12	4		
	Sinanodonta woodiana	97						11
	Atriana vexillum				4			
	Atactodea striata	5	1					
	Gafrarium tumidum	2525	1			5		
	Hippopus hippopus		1					
Crustacea	Litopenaeus vannamei				5			
	Thalamita admete	62						
	Thalamita crenata	56		12	1			
	Thalamita sima	71						
Echinoderms	Actinopyga caroliniana		1					
	Diadema setosum							135
	Holothuria aff.atra		8			2		
	Holothuria fuscocinerea		1					
	Holothuria lesson					60		
	Holothuria notabilis					2	125	
	Phyllophorus sp.					69	49	1
	Salmacis sphaeroides		50	58	95			
	Temnopleurus alexandri		3					
	Thelenota ananas			26	9	24		
	Thelenota anax					73	51	
	Tripneustes gratilla		851	57	289	7		
Gastropods	Canarium fusiformis			18	6			
	Canarium urceus			1510	62		410	1563
	Cymbiola vespertilio		9	25	17	1	1	

Chicoreus brunneus		5	11				
Conomurex luhuanus		173			1300		1
Conus marmoreus		19					
Cypraea mauritiana		32					
Lambis-lambis		155		10	141		
Pleuroploca trapezium		6					
Strombus sinuatus	14						
Strombus urceus	4						
Total number of individuals	2839	1318	1880	510	1688	636	1711
Total number of species	9	16	9	11	12	5	5
